# A New Method for Radiosynthesis of ^11^C-Labeled Carbamate Groups and its Application for a Highly Efficient Synthesis of the Kappa-Opioid Receptor Tracer [^11^C]GR103545

**DOI:** 10.2174/1874104500802010072

**Published:** 2008-07-16

**Authors:** B.W Schoultz, E Årstad, J Marton, F Willoch, A Drzezga, H.-J Wester, G Henriksen

**Affiliations:** 1 Department of Chemistry, University of Oslo, Norway; 2 Institute of Nuclear Medicine, University College London Hospitals NHS Trust, 235 Euston Road, London NW1 2BU, UK; and Hammersmith Imanet Limited, Hammersmith Hospital, Du Cane Road, London W12 0NN, UK; 3 ABX Biomedizinische Forschungsreagenzien GmbH, Radeberg, Germany; 4 Department of Radiology, Aker University Hospital, Aker Universitetssykehus HF, N-0514 Oslo, Norway; 5 Department of Nuclear Medicine, Klinikum rechts der Isar, Technische Universität München, Germany

## Abstract

^11^C-labeled carbamates can be obtained in a three-component coupling reaction of primary or secondary amines with CO_2_ and ^11^C-methylation reagents. [^11^C]Methyl-triflate mediated methylation of carbamino adducts provides the corresponding ^11^C-labeled carbamate groups in excellent yields under mild conditions (temperatures ≤ 40°C, 2 min reaction time). The utility of the method has been demonstrated by a highly efficient radiosynthesis of [^11^C]GR103545.

## INTRODUCTION

*In vivo* quantification of opioid receptors (ORs) and changes in their distribution and availability in the central nervous system (CNS) following neurodegenerative diseases, substance abuse and nociceptive signaling is crucial for precise diagnosis and therapy monitoring (for review see: Henriksen and Willoch, 2008 [1]). Three well-defined subtypes of cerebral ORs with different functional properties are known: μ-,δ- and κ-OR (Knapp et al., 1995) [2], Connor and Christie, 1999) [3]. A number of tracers have been developed for imaging of opioid receptors with positron emission tomography (PET) (Henriksen and Willoch) [1], Henriksen et al., 2006 [4], however only the δ-OR-selective [^11^C-methyl]naltrindole and the µ-OR-selective [^11^C]carfentanil depict single subclasses of the ORs in humans.

11C-labeled carbamate (-)-4-methoxycarbonyl-2-[(1-pyrrolidinylmethyl]-1-[(3,4-dichlorophenyl)acetyl-piperidine ([^11^C] GR103545) is a promising PET tracer for imaging of the κ-OR. Dynamic studies with [^11^C]GR103545 in baboons demonstrated high κ-OR affinity, excellent brain penetration, rapid uptake and wash-out kinetics, and low degree of non-specific binding (Talbot *et al.*, 2005 [5]).

The reported radiosynthesis of [^11^C]GR103545 relies on reduction of cyclotron produced [^11^C]carbon dioxide to form [^11^C]methanol, treatment with phosgene to produce [^11^C]methyl chloroformate, and subsequent reaction with the amine precursor to provide the 11C-labeled carbamate group (Ravert *et al.*, 1999 [6]; (Ravert *et al.*, 2002) [7]. Unfortunately, the method provides low radiochemical yields (RCY) in the range of 2 (Talbot *et al.* 2005) to 14% (Ravert *et al.*, 2002) [7]. Together with the large variations in the specific activity obtained (2564 mCi/µmol (Ravert *et al.*, 2002) [7], 150-495 mCi/µmol (Talbot *et al.* 2005 [5]), it is indicated that this method is capricious and thus effectively preventing human studies with [^11^C]GR103545.

Here we report a new method for radiosynthesis of ^11^C-carbamate groups based on *in situ* reaction of amines with carbon dioxide and subsequent ^11^C-methylation of the carbamino adduct. As the method only requires a single radioactive step, the entire process of labeling, purification and product formulation can readily be carried out using an automated synthesis module.

**Scheme 1:**
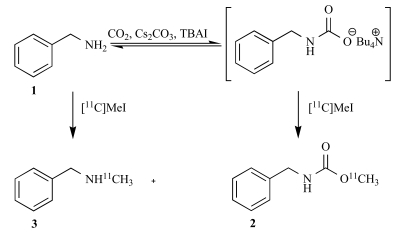
Reaction of benzylamine with CO_2_ to form the carbamino adduct and alkylation with [^11^C]CH_3_I.

In the initial part of the study, the carbamation of benzylamine **1** was formed by reaction with CO_2_ in the presence of Cs2CO3 and tetrabutyl ammonium iodide (TBAI). ^11^C-Methylation with [^11^C]CH_3_I under conditions similar to those reported for formation of non-radioactive carbamates (Salvatore *et al.,* 2001 [8]) provided carbamate **2** (Table **1**) in 18-36% radiochemical yield (RCY) [9]. Attempts to improve the radiochemical yield by heating the reaction mixture resulted in formation of N-[^11^C-methyl]benzylamine (**3**) [10] in addition to the desired [^11^C]carbamate **2**. The reduced chemoselectivity of the reaction at elevated temperature may be due to decomposition of the carbamino adduct and subsequent *N*-alkylation of the free amine as outlined in Scheme **1**.

Because of the apparent instability of the carbamino adduct at higher temperature, methylation with the more reactive [^11^C]methyl-triflate ([^11^C]CH_3_OTf) [11] was evaluated. Initial experiments with [^11^C]CH_3_OTf were performed under identical conditions to those used for alkylation with [^11^C]CH_3_I. Also in these experiments, the N-methylated side product **3** was observed when the reaction was carried out at elevated temperatures. We speculated that the large excess of iodide in the reaction mixture, resulting from addition of TBAI, may react with [^11^C]CH_3_OTf to form [^11^C]CH_3_I *in situ*. Substituting the phase-transfer catalyst with tetrabutyl ammonium triflate (TBAOTf) resulted in RCYs in the range of 69 ± 8 to 91 ± 5% within 2 min at 25 and 40°C, respectively (Table **1**). Notably, even at 40°C the reaction provided the [^11^C]carbamate **2** exclusively with no formation of *N*-[^11^C-methyl]-benzylamine (**3**). Thus, the results suggest that the high reactivity of [^11^C]CH_3_OTf enables alkylation to proceed to completion prior to decomposition of the carbamino adduct.

Using the optimized conditions for ^11^C-carbamate formation, *des*-carbamate-GR103545 [12] was converted to [^11^C]GR103545 in up to 91 ± 5% RCY (Table **2**). The carbamino adduct solution in DMF was found to effectively trap [^11^C]CH_3_OTf, with a volume of 100 μl sufficient to retain > 90% of the total activity introduced. Using the reduced volume (100 μl) of the precursor solution in preparative runs [13], [^11^C]GR103545 was obtained in 85 ± 6% isolated RCY, with a specific activity of 1792 ± 312 mCi/μmol and radiochemical purity of > 98%. The total synthesis time, including purification and formulation, was < 25 min after end-of-bombardment (n = 8). The short synthesis time, high specific activity and excellent RCY achieved with this method should facilitate evaluation of the κ-OR tracer [^11^C]GR103545 in clinical trials.

## CONCLUSION

[^11^C]MeOTf has been shown to rapidly methylate carbamino adducts of primary and secondary amines, providing the corresponding ^11^C-carbamate groups in excellent yields under mild conditions (temperatures ≤ 40°C, 2 min reaction time). The utility of the method has been demonstrated by a highly efficient radiosynthesis of [^11^C]GR103545. The method is suitable for automated routine production using synthesis modules compatible with good manufacturing practice.

## Figures and Tables

**Table 1:** Radiochemical Yield^a^ of *N*-benzyl-[^11^C]methyl Carbamate (2)^b^,^c^

Phase-Transfer Catalyst	Methylation Agent	T (°C)	Reaction Time (min)	RCY^a^
TBAI	[^11^C]MeI	25	5	36 ± 7
TBAI	[^11^C]MeI	40	5	27 ± 6
TBAI	[^11^C]MeI	50	5	21 ± 3
TBAI	[^11^C]MeI	70	5	18 ± 4
TBAOTf	[^11^C]MeOTf	25	2	69 ± 8
TBAOTf	[^11^C]MeOTf	25	5	78 ± 6
TBAOTf	[^11^C]MeOTf	35	2	82 ± 4
TBAOTf	[^11^C]MeOTf	40	2	91 ± 5

a Determined by analytical radio-HPLC

b Results represent the mean ± sd, n = 4

c To a solution of benzylamine (**1**) (2 mg, 18.6 µmol) in anhydrous DMF (500 µl) was added 3 molar equivalents of the TBA-reagent and 3 molar equivalents of Cs_2_CO_3_. CO_2_ gas (20 ml/min) was bubbled through the suspension for 1 h at room temperature. The ^11^C-alkylating agent was swept trapped in the reaction vial and allowed to react for the desired period of time.

**Table 2:** Radiochemical Yield^a^ of [^11^C]GR103454^b^,^c^

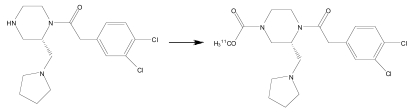		
T (°C)	Reaction Time (min)	RCY^a^
25	5	64 ± 5
35	5	72 ± 6
40	2	84 ± 4
40	5	91 ± 5

a Determined by analytical radio-HPLC

b Results represent the mean ± std, n = 5.

c To a solution of *des*-carbamate-GR103545 (3 mg, 8.4 µmol) in anhydrous DMF (500 µl) was added 3 molar equivalents of the TBA-reagent and 3 molar equivalents of Cs_2_CO_3_. CO_2_ gas (20 ml/min) was bubbled through the suspension for 1 h at room temperature. Subsequently, the ^11^C-alkylating agent was swept trapped in the reaction vial and allowed to react for the desired period of time.

## References

[1] Henriksen G, Willoch F (2008). Brain.

[2] Knapp RJ, Malatynska E, Collins N, Fang L, Wang JY, Hruby VJ, Roeske WR, Yamamura HI (1995). FASEB J.

[3]  Connor M, Christie MDJ (1999). Clin. Exp. Pharmacol. Physiol.

[4] Henriksen G, Willoch F, Talbot PS, Wester H-J (2006). Drug. Dev. Res.

[5] Talbot PS, Narendran R, Butelman ER, Huang Y, Ngo K, Slifstein M, Martinez D, Laruelle M, Hwang DR (2005). J. Nucl. Med.

[6] Ravert HT, Mathews WB, Musachio JL, Scheffel U, Finley P, Dannals RF (1999). Nucl. Med. Biol.

[7] Ravert HT, Scheffel U, Mathews WB, Musachio JL, Dannals RF (2002). Nucl. Med. Biol.

[8] Salvatore RN, Shin SI, Nagle AS, Jung KW (2001). J. Org. Chem.

[R9] 9To a solution of benzylamine (**1**) (2 mg, 18.6 μmol) in anhydrous DMF (500 μl) was added 3 molar equivalents of the TBA-reagent and 3 molar equivalents of Cs_2_CO_3_. CO_2 _gas (20 ml/min) was bubbled through the suspension for 1 h at room temperature. The ^11^C-alkylating agent was swept trapped in the reaction vial and allowed to react for the desired period of time. Aliquots were drawn and analyzed by analytical HPLC, which was performed using either a Chromolith RP18 4.6 × 100 mm reverse phase column (Merck) eluted with acetonitrile / 0.1 M ammonium formate (27.5:72.5, ^V^/_V_) mobile phase mixture at a flow rate of 5 ml/min (System A) or a Nucleosil 100 5 *μ*m C18 4.6 × 250 mm reverse phase column (CS-Chromatographie) eluted with acetonitrile / 0.1 M ammonium formate (55:45, ^V^/_V_) at a flow rate of 1 ml/min(System B). Both chromatography systems were fitted with a UV detector (Sykam Model S3210 set at 254 nm; Sykam, Fuerstenfeldbruck, Germany). For detection of radioactive compounds, a *γ*-ray detector (Bioscan Flow-Count fitted with a NaI(Tl) detector) was used in series with the UV detector.

[R10] 10*N*-[^11^C-methyl]benzylamine co-eluted on both analytical HPLC-systems with an authentic standard of *N*-methyl-benzylamine (Sigma-Aldrich)

[R11] 11Cyclotron produced [^11^C]CO_2_ was converted to [^11^C]CH_3_I by the catalytic gas-phase iodination reaction *via* [^11^C]CH_4_ (GE MeI MicroLab) and converted to [^11^C]CH_3_OTf by distillation through a column of AgOTf (1.6mm internal diameter, length 50 mm) impregnated on α-alumina

[R12] 12“Synthesis of *des-*carbamate-GR103.545 was adapted from that of Ravert *et al., *2002 [6] as follows: A solution of 1-[(3,4-Dichlorophenyl)acetyl]-4-(phenylmethyl)-2-[(1-pyrrolidinyl)methyl] piperazine (1.92 g, 4.3 mmol) in tetrahydrofuran-water 1:1 (%) (40 ml) and concentrated hydrochloric acid (4 ml) was hydrogenolysed under heterogenous catalytic conditions (atmospheric pressure, room temperature) in the presence of 10% palladium on charcoal (780 mg) for 5 h. The catalyst was removed by filtration and the filtrate was concentrated. Water (60 ml) was added to the residue and the solution was basified with 2M sodium carbonate (80 ml). The suspension was extracted with dichloromethane (5 x 100 ml). The organic layer was dried (Na_2_SO_4_) and evaporated under reduced pressure. The crude product was purified by column chromatography on silica gel (160 g) eluting with chloroform-methanol-NH_4_OH 9:1:0.1 (v/v/v). Yield: 1.29 g (84%), yellow oil. The racemic product was separated by means of preparative chiral HPLC: column: Chiracel OD-H, flow rate: 15 ml/min; λ = 254 nm; hexane-isopropanol-triethylamine 80:20:0.01 (v/v/v); retention time: peak 1: 8.77 min, peak 2: 15.34 min. Optical rotation: peak 1: [α]_D_^20^ = - 43.46 (c = 0.1, MeOH), peak 2: [α]_D_^20^ = + 44.13 (c = 0.1, MeOH). ― ^1^H-NMR (free base in CD_3_OD, 25°C):δ = 1.79 (m, 4H, pyrrolidine-3,4); 2.57 (m, 4H, pyrrolidine-2,5); 2.62-2.76 (m, 3H, 5-Hax, 3-Hax, 2-CH_2_-pyrr); 2.83-3.08 (m, 3H, 2-CH_2_-pyrr, 6-Heq, 5-Heq); 3.22 (dt, 1H, 6-Hax); 3.69 (m, 0.7H, 3-Heq); 3.78 (s, 2H, COCH_2_Ar); 3.99^mr^ (br t, 0.3H, 2-H); 4.32^mr^ (m, 0.3H, 3-Heq); 4.69 (br t, 0.7H, 2-H); 7.20 (m, 1H, Ph-C6); 7.45 (m, 2H, Ph-C2, Ph-C5)

[R13] 13Preparative HPLC was performed using a Chromolith RP18 10 × 100 mm reverse phase column (Merck) eluted with acetonitrile / 0.1 M ammonium formate (27.5:72.5, ^V^/_V_) at 10 ml min. In-line HPLC detectors included a UV detector (Sykam) set at 254 nm and a γ-ray detector (Bioscan Flow-Count fitted with a PIN detector). The fraction containing the product (*t*_r_ = 2.8 min) was diluted with water and applied to a Sep-Pak C18 solid phase extraction cartridge (Waters). The cartridge was washed with 10 ml of water before eluting the product with EtOH. The labeled product co-eluted with an authentic standard of GR103.545 on the two analytical HPLC systems [9] (system A: *t*_r_ = 1.8 min; system B: *t*_r_ = 4.2 min)

